# Mass Smallpox Vaccination and Cardiac Deaths, New York City, 1947

**DOI:** 10.3201/eid1005.040119

**Published:** 2004-05

**Authors:** Lorna E. Thorpe, Farzad Mostashari, Adam M. Karpati, Steven P. Schwartz, Susan E. Manning, Melissa A. Marx, Thomas R. Frieden

**Affiliations:** * New York City Department of Health and Mental Hygiene, New York, New York, USA; † Centers for Disease Control and Prevention, Atlanta, Georgia, USA

**Keywords:** Adverse effects, smallpox prevention, mass vaccination, ischemic heart disease, mortality, vaccinia

## Abstract

In April 1947, during a smallpox outbreak in New York City (NYC), >6,000,000 people were vaccinated. To determine whether vaccination increased cardiac death, we reviewed NYC death certificates for comparable periods in 1946 and 1948 (N = 81,529) and calculated adjusted relative death rates for the postvaccination period. No increases in cardiac deaths were observed.

Smallpox was successfully eradicated in 1980 after a global vaccination campaign by the World Health Organization. After the terrorist events of September and October 2001, the U.S. government initiated a campaign to immunize the American military and civilian first-responders in the event of an intentional release of the smallpox virus (*1*). From December through April 2003, smallpox vaccine was administered to 29,584 civilians and 365,000 military personnel nationwide (*2*,*3*). By March 28, four nonfatal and three fatal myocardial infarctions (MIs) had been reported. Whether these ischemic deaths were vaccine-associated or co-incidental is unclear.

To ascertain whether cardiac deaths increased after a large 1947 smallpox vaccination campaign in New York City (NYC), we examined death certificates from a 4-month period in 1947 as well as from comparable periods in 1946 and 1948. Key findings were published in an earlier article (*4*). We provide full results and additional methodologic detail here.

## The Study

From April 4 through May 2, 1947, 6.35 million New Yorkers were vaccinated with the NYC Board of Health vaccinia strain (*5*). We used newspaper accounts and NYC Department of Health records to estimate the number of adults vaccinated on each of the 29 days (*5*). Since all of the 2003 cardiac events occurred from 4 to 17 days after vaccination, the 1947 vaccination numbers were divided equally across the same 14-day period to calculate the person-time at risk for potential cardiac death. On the basis of these estimates, we identified the 2- and 4-week “peak” risk periods in 1947.

We obtained all death certificates issued in NYC for the 4-month period between March and June, 1946–1948, from the NYC Municipal Archive. Cause of death was coded according to the International Classification of Diseases, 5th Revision (ICD-5) (*6*). We abstracted the date of death, age of decedent, and ICD-5–coded primary and other cause of death into an electronic database. We defined cause of death as “cardiac” if the ICD-5 codes for either cause included pericarditis (090), acute endocarditis (091), chronic endocarditis (092), myocardial disease (093), coronary artery diseases (094), and other disease of the heart (095).

We compared daily death rates during the postvaccination risk periods with rates at other times during the study period. We used Poisson regression, a generalized linear model appropriate for analysis of discrete data, to model counts of cardiac deaths (*7*). Counts were used instead of rates, as NYC’s population remained relatively constant during the study’s 3-year timeframe. We also adjusted for temporal trends in the data: a long-term trend from 1946 to 1948 (defined by weeks since January 1, 1946) and a seasonal trend between March and June (defined by days since March 1 for any given year). Secular trends were modeled with linear and quadratic terms. The main model included all cardiac deaths as the outcome variable and a dichotomous “exposure” variable indicating whether the death occurred during the 2-week risk period. Additional models examined subsets of cardiac disease and all-cause death as outcomes, as well as adjusting for noncardiac death volume.

An a priori power analysis found that the model had >90% power to detect a 5% increase in cardiac fatalities in the at-risk period. While this power would be more than sufficient to detect an excess of 2 deaths in 29,584 civilians (approximately 400 deaths in the 1947 NYC population of 6,000,000), it would not be able to detect very small elevations in risk.

**Figure 1:**
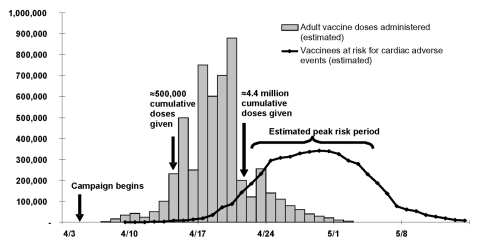
Adult vaccination doses administered and estimated person-time at risk for fatal cardiac adverse effects, New York City, 1947.

At the height of the 1947 vaccination campaign, from April 17 to April 21, 500,000 to 1 million people were vaccinated daily (Figure 1). The 2-week at-risk period in 1947 was estimated to be April 22 to May 5, which encompassed 84% of the projected at-risk person-time for adverse cardiac complications. The 4-week period was identified as April 16 to May 13 and included 99% of the at-risk person-time.

**Figure 2:**
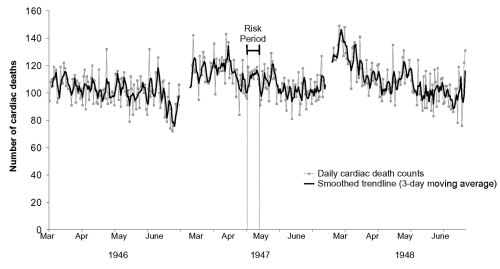
Daily deaths from cardiac causes, New York City, March to June, 1946–1948.

During the months under review in 1946–1948, 81,529 death certificates were recorded, including 519 (0.6%) records with an illegible cause of death. Of the remaining 81,010 records, 48% had heart disease listed as a cause of death. A total of 9,112 (11%) specifically referred to coronary artery or atherosclerotic disease. The number of daily deaths from heart disease in the months of March to June of 1946, 1947, and 1948 ranged from 72 to 149, with an increasing long-term trend and decreasing seasonal trend (Figure 2). In the 2-week estimated risk period in 1947, 1,545 cardiac deaths occurred of 3,156 total deaths (average 110 deaths per day, range 91–119 deaths) (Table 1).

**Table 1:** Death counts by cause of death and postvaccination exposure period

Timeframe	All deaths	Cardiac deaths	Atherosclerotic deaths
Mar–Jun 1946	26,256	12,340	2,861
Mar–Jun 1947	27,484	13,352	3,075
Mar–Jun 1948	27,774	13,458	3,175
2-week exposure period	3,156	1,545	280

In the main regression model (Table 2), no independent association was found between cardiac deaths and the 2-week estimated risk period. The findings remained nonsignificant when the model was restricted to those 50 to 64 years of age and when adjustments for noncardiac deaths were made (rate ratio 1.01; 95% confidence interval 0.95 to 1.06). Additional analyses examining different outcomes (all deaths, atherosclerotic deaths, or deaths due to myopericarditis) did not show any significant increase in deaths, nor did expanding the estimated risk period to 4 weeks.

**Table 2:** Rate ratio (RR) and 95% confidence interval (CI) of cardiac death rates comparing postvaccination to reference periods,^a^ New York City, March–June, 1946–1948

Outcome	Postvaccination period	RR (95% CI)
All cardiac deaths (ICD-5 090–095)	April 22–May 5	1.01 (0.96 to 1.07)
50- to 64-year-olds only		1.05 (0.95 to 1.15)
Atherosclerotic cardiac deaths (ICD-5 094)	April 22–May 5	1.06 (0.97 to 1.16)
50- to 64–year-olds only		1.00 (0.86 to 1.15)
Myopericarditis deaths (ICD-5 090, 093)	April 22–May 5	1.00 (0.94 to 1.07)
All deaths	April 22–May 5	1.00 (0.97 to 1.04)
All cardiac deaths (ICD-5 090–095)	April 16–May 13	0.99 (0.95 to 1.04)

^a^All models are adjusted for long-term temporal and seasonal trends.

## Conclusions

Our analysis found no significant increase in reported cardiac deaths after the 1947 mass smallpox vaccination campaign in NYC. The campaign was unique in terms of the number of people vaccinated in one area in a short period. The high intensity and coverage of the vaccination campaign permitted a focused cardiac death assessment.

Recent reports of cardiac deaths after smallpox vaccination have raised concerns regarding the safety of the current vaccination initiative. The NYC Board of Health vaccinia strain used today is the same as was used in 1947 (*5*). As described in our prior publication (*4*), vaccinia is a DNA virus with limited antigenic variability (*8*), and antigenic shifts are unlikely. Regarding the vaccinated population, major risk factors, such as smoking and hypertension, were more widespread in 1947 than they are at present (*9*–*11*), and the death rate due to heart disease was nearly three times higher (*11*). If, as the 2003 cardiac fatalities suggest, cardiac risk factors increase vaccine-associated death rates, we should have seen an even-greater cardiac mortality risk in 1947.

Our analysis has some limitations. First, this analysis was ecologic, and we had no information on the vaccination status of decedents. More than 80% of the NYC population was vaccinated within the 4-week period, however, which minimizes the risk of faulty ecologic inference. The campaign urged all New Yorkers to get vaccinated, irrespective of age, health, or pregnancy (*5*), and the likelihood of systematic bias that would mask an association is small.

Second, death certificate information may have been incomplete or inaccurate. We extracted ICD codes for >99% of hardcopy death certificates, and missed codes were unlikely to affect the findings of the study. We also have no reason to believe that cardiac deaths were systematically misclassified in the peak risk exposure period as compared with other times. ICD-5 heart disease codes and later ICD revisions have been assessed to have a high comparability ratio, from 0.98 to 1.01 (*6*).

Third, assumptions pertaining to a Poisson distribution may not be appropriate for these cardiac death data (*11*). However, no biologically plausible concern existed for underdispersion, and goodness-of-fit statistics suggested adequate fit. Null findings of the study reduce concern for overdispersion, which could have otherwise potentially caused us to report an association that was not causal.

Finally, although the survey had substantial statistical power to detect small increases in cardiac deaths in the estimated at-risk period, extremely small increases may not have been detectable. In a large population, even small elevations in risk will produce a sizable absolute number of deaths. In light of this limitation, common to all observational studies, these findings should be interpreted in the context of other study findings.

Any one study will not likely be able to definitively rule out a causal relationship between cardiac deaths among recent vaccinees and the vaccine itself, but findings from our study provide some reassurance that the current smallpox vaccination program is unlikely to increase risk for death from coronary disease. In 1947, the commissioner of health of NYC reminded his peers, “Whenever a large-scale vaccination program is undertaken, there is always the possibility that there may be some unfortunate complications…. In New York City, there are thousands of people who become ill, and about two hundred of them die every day. Since practically every person in New York City had a recent vaccination, it was inevitable that some of them would become ill and would die. Vaccination does not stop the normal course of events. Neither should vaccination be blamed for a death from cerebral hemorrhage, nephritis, or coronary occlusion (*5*).”
